# On the Influence of Mercury on the Functions of the Uterus

**Published:** 1829-04-01

**Authors:** 


### VIII.

### HOPITAL DES VENERIENS.

ON THE INFLUENCE OF MERCURY ON THE FUNCTIONS OF THE UTERUS. By ALEXANDER COLSON, M.D. Formerly House-Surgeon of the Hôpital des Veneriens, &c.

The functions of the uterus, both in a state of menstruation and impregnation, are often disturbed by strong medicinal agents; and as mercury is used in this country, to a greater extent than in any other, excepting, perhaps, America, it may be interesting to inquire, what are the effects of this medicine on the uterine functions. Such inquiry can best be prosecuted in a Lock-Hospital; and it is to this subject Dr. Colson has directed his attention.

The uterine derangements produced by mercury, he observes, are, mænorrhagia, amenorrhœa, and abortion. These effects Dr. C. illustrates by a series of cases, one or two in each class we shall briefly state.

1. *Mænorrhagia.* A young woman, 18 years of age, was received into hospital on the 17th October, for the cure of vegetations about the labia. She had been regular in her menses for four years previously. She was put on the use of Van Sweiten's liquor, (solution of oxymuriate of mercury) and eight days afterwards complained of pains in the abdomen, but especially in the loins and hypogastrium. The menses came on eight days in advance, and they continued to flow during more than two months that she remained in hospital. The mercury had no effect on the vegetations. They were excised—and in eight days afterwards, the patient was discharged cured. Four or five other cases are detailed, shewing the mænorrhagic influence of mercury; but the above is a sufficient illustration.

2. *Amenorrhœa.* C. Virginie, aged 19 years, had contracted a syphilitic complaint, three months after she went on the town. She entered the Venereal Hospital, and was put on the use of the oxymuriate, which affected her mouth. Hitherto she had been regular in her courses; but now they stopped, and continued so all the time she was at the hospital. Two applications of leeches to the vulva were made in vain, as well as sinapisms, pediluvia, &c. She continued nearly three months in hospital, and left it in a state of amenorrhœa and general bad health, though the syphilitic complaint was cured. In about six weeks after she left the hospital, the menses re-appeared. Six other cases, of a similar kind, are detailed by the author.

3. *Abortion.* A female, aged 24 years, six months pregnant, was received into the hospital, in the month of October, for the cure of gonorrhœa. She was subjected to *mercurial frictions!* At the end of a fortnight of this treatment, she complained, that she no longer felt the motions of the child, which previously were very distinct. She also complained of a sense of weight at the lower part of the abdomen, and in the groins. This state continued three or four days, and ended in the abortion of a fœtus dead, and apparently of about seven months.—ARCHIVES.

This case is followed by five others, where the premature expulsion of the fœtus was reasonably referrible to the exhibition of mercury. The author remarks, that miscarriages are very common in the Venereal Hospital of Paris, though this accident is usually attributed to the syphilitic complaint, and not to the mercury employed for its cure. Dr. C. is of a different opinion, and attributes the abortions to the mercury. We do not deem it necessary to enter into the reasonings adduced by our author, respecting the modus agendi of mercury on the fœtus. The facts are of more value than the arguments; and, as far as these facts go—and they are not very few in number—they offer fair testimony of the injurious effects of mercury on the functions of the uterus. How far the particular mode of administration in the Parisian Hospitals, may increase these bad effects of mercury, we are not able to determine, but this short notice of a lengthy memoir may convey some useful precautionary hints to the English practitioner.

